# Novel Patchouli Alcohol Ternary Solid Dispersion Pellets Prepared by Poloxamers

**Published:** 2015

**Authors:** Jin-Bin Liao, Yong-Zhuo Liang, Yun-Long Chen, Jian-Hui Xie, Wei-Hai Liu, Jian Nan Chen, Xiao-Ping Lai, Zi-Ren Su

**Affiliations:** a *School of Chinese Materia Medica, Guangzhou University of Chinese Medicine, Guangzhou 510006, PR China. *; b *Guangdong Second Province Hospital of Traditional Chinese Medicine, Guangzhou 510095, P. R. China. *; c *Dongguan Mathematical Engineering Academy of Chinese Medicine, Guangzhou University of Chinese Medicine, Dongguan 523808, PR China.*; d *Institute of Higher Education, Guangzhou University of Chinese Medicine, Guangzhou 510405, PR China.*

**Keywords:** Ternary solid dispersion pellets, Patchouli alcohol, Poloxamers, Eutectic mixtures, Closed in-vitro dissolution test method

## Abstract

The present study investigates the possibility of using poloxamers as solubility and dissolution rate enhancing agents of poorly water soluble bioactive constituent patchouli alcohol (PA) that can be used for the preparation of immediate release pellets formulation. Two commercially available grades poloxamer 188 (P 188) and poloxamer 407 (P 407) were selected, and solid dispersions (SDs) containing different weight ratio of PA and poloxamers, and the combination of P 188 and P 407 as dispersing carriers of ternary solid dispersions (tSDs) were prepared by a low temperature melting method and solidified rapidly by dropping into the 10-15 °C condensing agent atoleine. Both PA/P 188 and PA/P 407 binary solid dispersions (bSDs) could remarkably promote the dissolution rate of PA, increasing approximately 16 times in bSDs with poloxamers in comparison with pure PA within 180 min. P188 contributed to a faster dissolution rate than P 407, however, P 407 had a better solubility. It is interesting to note that the incorporation of P 188 in PA/P 407 bSD pellets could strongly enhance the dissolution rate of PA. DSC and FTIR were used to explore the characteristics of PA-SD pellets. The enhancement of dissolution from the SDs may be attributed partly to the reduction in particle size in PA crystalline due to the formation of eutectic system with poloxamers. Moreover, a simple, accurate* in-vitro* dissolution test method for volatility drug was established, and the process of PA-SD pellets preparation was simple, rapid, cost effective, uncomplicated and potentially scalable.

## Introduction

**Figure 1:**
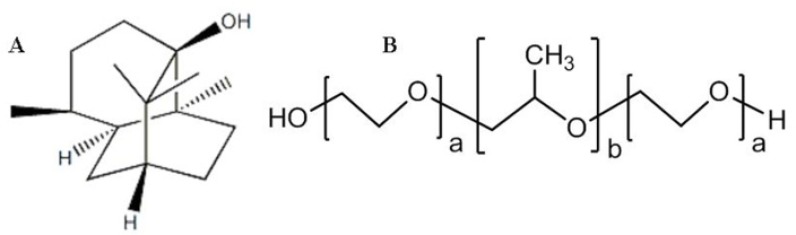
(A) Chemical structure of PA. (B) Chemical structure of Poloxamers P 188 (a=75-85, b=25-30) and P 407 (a=95-105, b=54-60).

Patchouli alcohol (PA) (Figure 1A), a tricyclic sesquiterpene, is a major liposoluble bioactive constituent extracted from the leaves of *Pogostemon cablin* Benth, which has been used as a component herb of traditional herbal prescriptions in China as an aromatic stomachic to improve impairment of the digestive system. PA exhibits a variety of activities including anti-*Helicobacter *pylori (1), anti-inflammatory (2, 3), anti-influenza (4), cognitive enhancing, learning impairment attenuating and neuroprotective (5, 6). PA is a white, crystalline solid at room temperature with a melting point of 54.3 °C. It is readily soluble in organic solvents and lipids but practically insoluble in water, which could be the rate determining step for poorly water-soluble active pharmaceutical ingredients’ efficient absorption (7). 

Various approaches can be used for the enhancement of solubility and dissolution rate of poorly water soluble drugs like PA, such as particle size reduction (8), using salt formation of ionizable drugs (9), solubilization in surfactant systems (10), and complexing drugs by using cyclodextrins (11), or preparation of solid dispersions (12-15). However, particle size reduction may not be desirable in situations because of handling difficulties and poor wettability for very fine powders. Salt formation is not feasible for neutral compounds and the synthesis of weak acid and weak base salts may not always be practical (16). Moreover, an increased dissolution rate in the gastrointestinal tract may not be achieved in many cases because of the possible reconversion of salts into aggregates of their respective acid or base forms (16). Some researchers have tried to improve the solubility and dissolution rate of PA by using self-microemulsifying drug delivery systems (17). However, the solubilization of drugs in organic solvents or in aqueous media by the use of surfactants and cosolvents leads to liquid formation that is usually undesirable from the viewpoints of patient acceptability (16). 

Solid dispersions are one of the most promising strategies for promoting dissolution, as they overcome the limitations of previous techniques. Once the solid dispersion is exposed to aqueous media and the carrier dissolves, the drug is released as very fine, colloidal particles, thus resulting in higher surface area and consequently enhances dissolution rate and bioavailability of poorly water soluble drugs (18, 19). Moreover, no energy is required to break up the crystal lattice of a drug in the amorphous state during dissolution process (20) and its solubility as well as wettability may be increased by surrounding hydrophilic carriers (21).

Poloxamers (P 188 and P 407) (Figure 1B) are hydrophilic nonionic poly(oxyethylene)-poly(oxypropylene)-poly(oxyethylene) block copolymer that have been widely used as wetting and solubilizing agents, and surface adsorption excipients (22-24). There are white or almost white, waxy powders, microbeads or flakes. Very soluble in water and in alcohol, practically insoluble in light petroleum (50-70 °C). Melting point, about 50 °C (25). HLB value, >24 and 18-23 for P 188 and P 407, respectively (26). It is widely used in pharmaceutical formulations as an emulsifying or solubilizing agent. Some reports demonstrated that P 188 and P 407 could increase the solubility of poorly soluble drug significantly via the preparation of solid dispersion by melting method (27, 28). 

In order to improve the solubility and oral bioavailability of PA, the present study was undertaken to investigate the possibility of preparing solid dispersions by a low temperature melting method with poloxamers. The effect of two poloxamers and their combination as dispersing carries on the dissolution rate of PA were explored. And the establishment of a simple, accurate *in-vitro* dissolution test method for volatility drug was also evaluated. Differential scanning calorimetry (DSC) and Fourier transform infrared spectroscopy (FTIR) were used to explore the characteristic of PA-SD pellets and demonstrate the value in preparing eutectic systems for improving the dissolution rate of PA.

## Experimental

*Materials*

PA (98.6%) was extracted and purified from* Pogostemon cablin* (Blanco) Benth. (Labiatae) as previously described (3). Poloxamers F-grades (Lutrol^®^F68 and Lutrol^®^F127) were obtained from BASF Aktiengesellschaft (Ludwigshafen, Germany). Demineralized water was used in all experiments. All reagents were of analytical grade.

*Preparation of PA-SD pellets*

PA and poloxamers were mixed in different weight ratios and melted in a glass evaporating dish immersed in a water bath at 70 °C with continuous stirring to obtain a homogeneous dispersion. The melted mixture was then solidified rapidly by dropping into the 10-15 °C condensing agent atoleine, and the dropping distance was 5-10 cm, dropping rate 30 drops min^-1^. Took out dropping pellets after 30 min, and absorbed the cold condensing agent adhering to the surface of SD pellets.

*Determination of PA*

Gas chromatograph analyses were performed on a model GC (Varian 3900, USA) equipped with a split/splitless capillary (1:20) injector and a flame ionization detector. Analytical separation was achieved on a 007-225 capillary column (30 m × 0.25 mm × 0.25 μm, Phenomenex, USA). Nitrogen was used as carrier gas (with a constant flow rate of 1 mL/min). The air, hydrogen and auxiliary gas (N_2_) pressures for detector were kept 350, 30 and 30 mL/min, respectively. Temperature setting was as follows: injector, 280 °C; detector, 280 °C. The oven temperature was held at 110 °C for 2 min, then programmed to 240 °C at 20 °C/min, and held for 2 min at 240 °C. The peak areas were used to calculate the PA content. 

*Disintegration test*

The disintegration time of PA-SD pellets was determined with 900 mL of distilled water at 37 ± 0.5 °C using disintegration apparatus (ZB-1C, Tianjin, China) modified with a 0.42 mm wire mesh at both ends of the tubes. Six PA-SD pellets from each formulation were placed on the base wire mesh in the tube. Disintegration time was recorded when the PA-SD pellets were completely disintegrated and passed through the mesh.

*In-vitro* *release studies*

Dissolution experiments were performed in Dissolution Apparatus II (USP, paddles) (ZRS-8G dissolution apparatus, Tianjin, China). Samples containing approximately 40 mg of PA were added to 900 ml distilled water at 37 ± 0.5 °C with 100 rpm rotation rate. At predetermined time intervals, 4 mL of the sample was withdrawn and the same volume of fresh dissolution medium was supplemented after each point to keep constant volume. The collected samples were filtered through 0.45 µm filter. Initial sample volume of 2 mL was discarded and final 2 mL was collected, which was placed into a centrifuge tube and 2 mL octadecane solution (dissolved in ethyl acetate, concentration was about 45 µg/mL, as internal standard solution) was added. The solution was vortexed for 10 min and the mixture solution was centrifuged at 8000 rpm for 10 min. 1 µL of supernatant fluid was then analyzed by GC. The dissolution profiles of formulations with and without poloxamers were compared to confirm the prominent effect of combined carriers. Different ratios of PA, P 188 and P 407 were also investigated to screen the optimal formulation. Dissolution experiments were performed in triplicate and the average dissolution profiles, and standard deviations were calculated.

*Evaluation of open and closed in-vitro dissolution test methods for volatility PA*

Dissolution profiles of solid dispersions of PA in P 407 at the ratio of 1/5 under open/closed system conditions were comparatively investigated. The sample collection and determination were the same as described above. 

*Open system*

The open *in-vitro *dissolution test system was Dissolution Apparatus II (USP, paddles) (ZRS-8G dissolution apparatus, Tianjin, China) without any modification, which was an open system.

*Closed system*

The closed *in-vitro *dissolution test system was a modification of the Dissolution Apparatus II (USP, paddles) (ZRS-8G dissolution apparatus, Tianjin, China). A plastic cap was attached at top of the dissolution vessel using vaseline to form closed condition.

Comparison of two data sets was performed by paired Student’s t-test. *p *≤ 0.05 was considered as significantly different.

*Differential scanning calorimetry (DSC)*

DSC analyses were performed using a Shimadzu DSC-60 (Shimadzu, Japan) differential scanning calorimeter. Approximately 5 mg of sample in an aluminium standard pan was heated from 30 ºC to 100 ºC at a scanning rate of 5 ºC/min. An empty aluminum pan was used as reference.

*Fourier transform infrared spectroscopy (FTIR)*

The FTIR spectra were obtained on a Nicolet Avatar 330FT-IR spectrometer (Thermo Electron Corporation, Massachusetts, USA). The spectra of solid dispersions and physical mixtures of PA and poloxamers were mixed with dry KBr and measured in the spectral range from 400 to 4000 cm^-1^ with a spectral resolution of 4 cm^-1^ at room temperature. Thirty-two scans were taken for each sample.

## Results and Discussion

*Preparation of PA-SD pellets*

The resultant PA-SD pellets characterized by perfect appearance and high smooth roundness, which would facilitate the compliance of patients in medicine taking. And the preparation method is relatively low-cost, no residual solvent and easily scalable manufacturing processes.

*Disintegration* *time*

The results of disintegration test, obtained with the different formulations, are shown in Table 1. The disintegration time for all formulations of PA-SD pellets was within the range of 8-96 min. The incorporation of P 188 in PA/P 407 bSD pellets could significantly decrease the disintegration time, suggesting that P 188 could serve as a good disintegrant. In addition, it was observed that as the amount of P 188 in the tSD pellets increased, the disintegration time was reduced accordingly. The results were found to be related to the dissolution rate of PA-SD pellets. As the time taken for disintegration reduced, the dissolution rate of the PA-SD pellets increased.

**Table 1:** Disintegration time of the PA-SD pellets

**Formulation**	**Disintegration time (min) mean ± SD**
1/1 w/w bSD P 407	32.17 ± 6.40
1/3 w/w bSD P 407	68.50 ± 17.17
1/5 w/w bSD P 407	33.33 ± 1.75
1/1/1 w/w/w tSD	11.17 ± 2.48
1/3/1 w/w/w tSD	19.67 ± 5.01
1/5/1 w/w/w tSD	22.00 ± 3.41
1/5/3 w/w/w tSD	19.67 ± 4.41
1/5/5 w/w/w tSD	12.83 ± 3.25

*Evaluation of open and closed* *in-vitro dissolution test method* *s* *for volatility PA*

**Figure 2:**
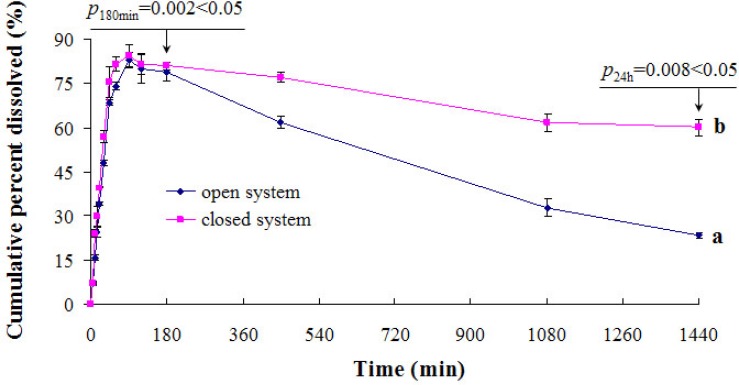
The effect of open/closed systems on dissolution profiles of (a) open system, (b) closed system. Each point represents the mean ± SD (*n* = 3).

This study reports the statistical evaluation of the performances of open and closed *in-vitro* dissolution test methods in terms of volatility losses for the determination of PA. Statistical evaluation of the data was done according to paired students’t test at a confidence level of 95%. As seen from Figure 2, open* in-vitro *dissolution test procedures gave lower results compared to the closed one. There were statistically significant difference within 180 min or 24 h. Results from the closed* in*-*vitro* dissolution test method were assumed to be accurate, and its application* in-vitro* dissolution test is highly recommended for the analysis of samples with volatility. 

*Effects of PA/poloxamers ratios on dissolution rate of PA*

**Figure 3:**
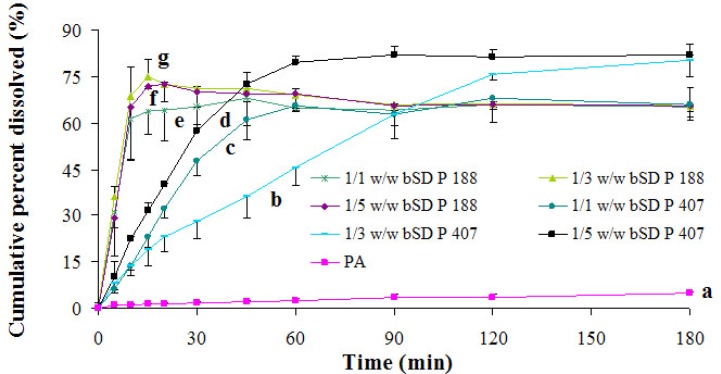
*In* *-* *vitro *release profiles of bSD pellets at different PA/poloxamers ratios. Each point represents the mean ± SD (*n* = 3).

The dissolution profiles of pure PA and drug from bSDs pellets containing various amounts of each of the two carrier polymers (P 188 and P 407) are shown in Figure 3. PA powders exhibited a poor dissolution rate with only 5% drug dissolved after 180 min (Figure 3a). Nevertheless, it was observed that the dissolution rate was increased by approximately 16 times in bSD pellets with poloxamers in comparison with pure PA within 180 min. The increase in dissolution rate of PA from bSD pellets might be attributed to various factors, such as a reduction in the drug crystallinity, simple eutectic mixture and molecular dispersion of drug in poloxamers. The solubility of PA from bSD pellets varied depending on the ratio of PA/poloxamers. With the ratios increasing, the solubility of PA elevated correspondingly, indicating that the increase in solubility could be achieved by increasing the amount of poloxamers in SD pellets.

However, as PA/P188 increasing to 1/5 (Figure 3f), the dissolution rate of PA slightly declined compared to bSD pellets with PA/P188 ratio of 1/3 (Figure 3g). Hence, it was possible that larger amount of P188 in bSD pellets provided better conditions for gel layer formation when in contact with water, which consequently acted as a diffusion barrier and delayed drug release. Interestingly, the bSD pellets with PA/P407 ratio of 1/3 (Figures 3b) showed unusually lower dissolution rate than either 1/1 or 1/5 ratio (Figures 3c and 3d), similar findings have been reported by Chutimaworapan *et al.* (29). The rate of drug dissolution is greatly influenced by disintegration, and a fast compact disintegration is desirable to increase the particle surface area and hence enhance drug release (30). As shown in Table 1, the bSD pellets with PA/P 407 ratio of 1/3 showed longer disintegration time than both 1/1 and 1/5 ratios, suggesting that the lower dissolution rate of bSD pellets with PA/P407 ratio of 1/3 might be attributable, at least in part, to the longer disintegration time. However, further study on the mechanism underlying the disintegration of PA-SD pellets is required.

It was noticeable that the bSDs containing P 188 contributed to a faster dissolution rate compared to P 407, which could be attributed to its lower molecular weight and higher proportion of hydrophilic polyoxyethylene segment, *i.e*. higher HLB value. However, bSDs containing P 407 had a better enhancement in drug solubility with respect to P 188.

*Effects of poloxamer 188 on dissolution rate of PA*

**Figure 4:**
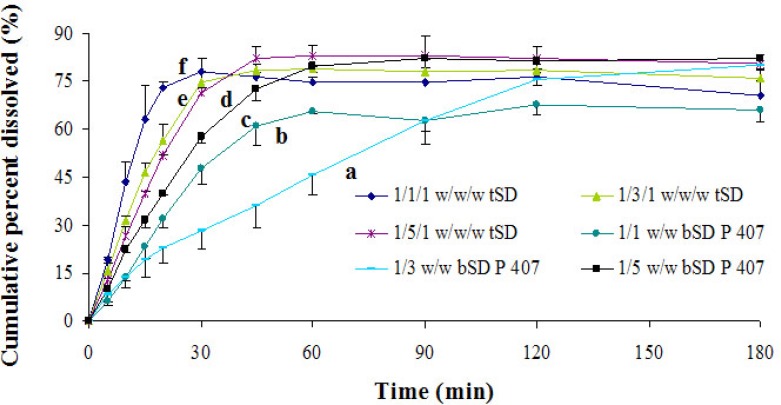
The dissolution profiles of bSD pellets at different PA/P 407 ratios and tSD pellets at different PA/P 407/P 188 ratios. Each point represents the mean ± SD (*n* = 3).

In order to further promote dissolution behavior of PA, a novel ternary solid dispersion systems consisting of PA, P 188 and P 407 were investigated. As shown in Figure 4, a faster dissolution rate of PA was achieved with the incorporation of P 188 in PA/P 407 bSD pellets compared to binary PA/P 407 systems at different ratios within 180 min. Mura *et al.* have found that P 188 present on surface could decrease the surface tension dissolution between media and drug particle and hence bringing about additional wettability and solubilizing effect (31). Serajuddin *et al.* also found that drug could be dispersed into mostly submicron size particles under the action of poloxamer 188, which could further facilitate the dissolution (32). As shown in Table 1, when comparing tSD pellets and PA/P 407 bSD pellets, a faster disintegration of PA was observed after the incorporation of P188, and the resulting rapid disintegration time of tSD pellets might result in an increased dissolving surface area of the drug and thereby an increased dissolution rate.

*Effects of incorporated poloxamer 188 content on dissolution rate of PA*

**Figure 5:**
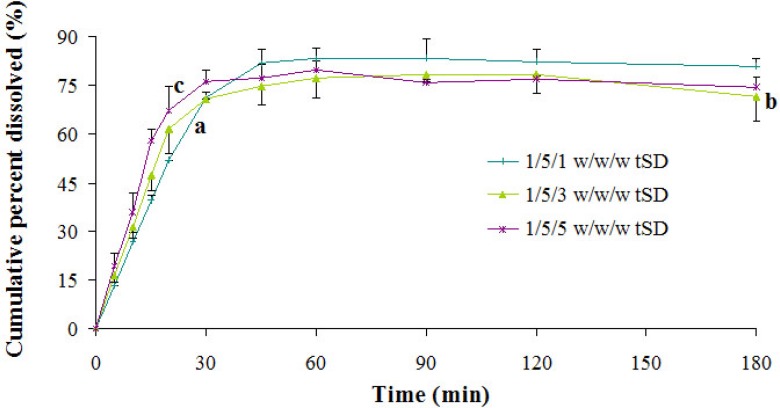
*In* *-* *vitro *release profiles of tSD pellets at different PA/P 407/P 188 ratios. Each point represents the mean ± SD (*n* = 3).

Effect of the content of P 188 in ternary systems on PA dissolution was investigated by comparing three different ratios of PA/P 407/P 188 (Figure 5). Judging from the dissolution results, the dissolution rate of PA in ternary systems was directly proportional to the content of P 188 within 30 min, which might be partially attributable to the reduction of disintegration time. However, after 30 min the dissolution rate of PA decreased at higher level (after 1/5/1 ratio). P 188 always existed in an amphiphilic structure, which possessed the properties to self-assemble into micelles in aqueous solution when the concentration was above the critical micellar concentration (CMC) (33). PA might be entraped by micelles formed by poloxamers, which became an obstacle to its further dissolution.

*DSC analysis*

A eutectic solid is a condensed phase that is formed when a specific composition of two miscible liquid phases is co-solidified at a specific temperature, resulting in a crystalline microstructure that has a lower melting temperature relative to that of either pure constituent (34). DSC is a fast, useful, and reliable analytical tool to detect and investigate the eutectic compound (35). 

**Figure 6:**
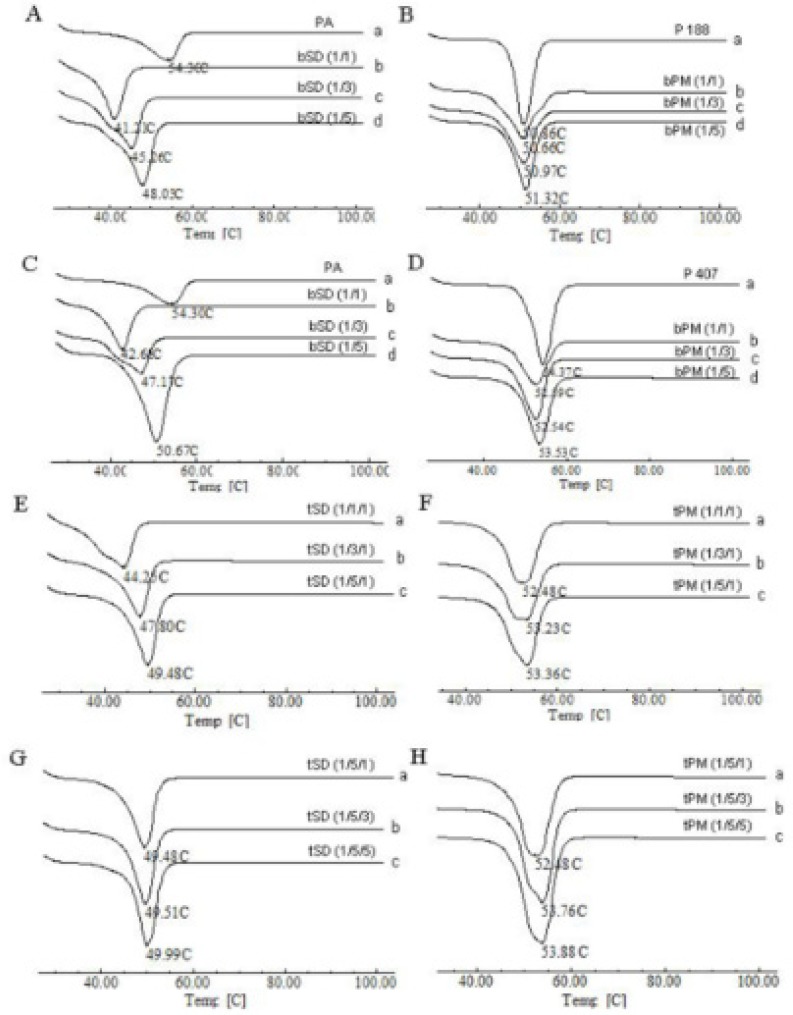
DSC curves of PA, P 407, P 188, physical mixtures and SD pellets.

**Figure 7:**
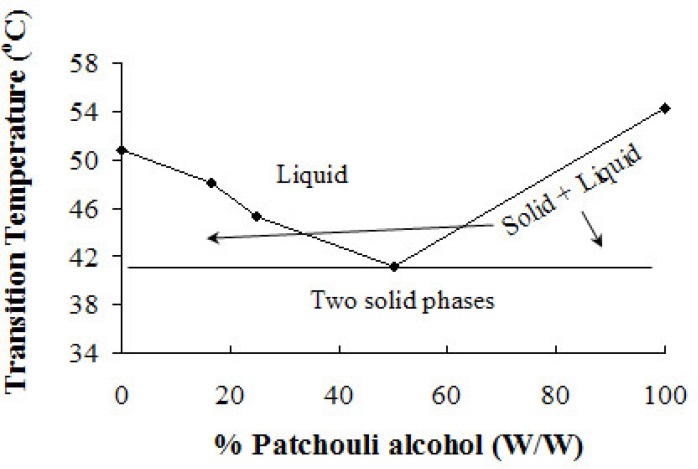
Binary phase diagram of PA/P 188 bSD pellets. The lines have no theoretical significance.

**Figure 8:**
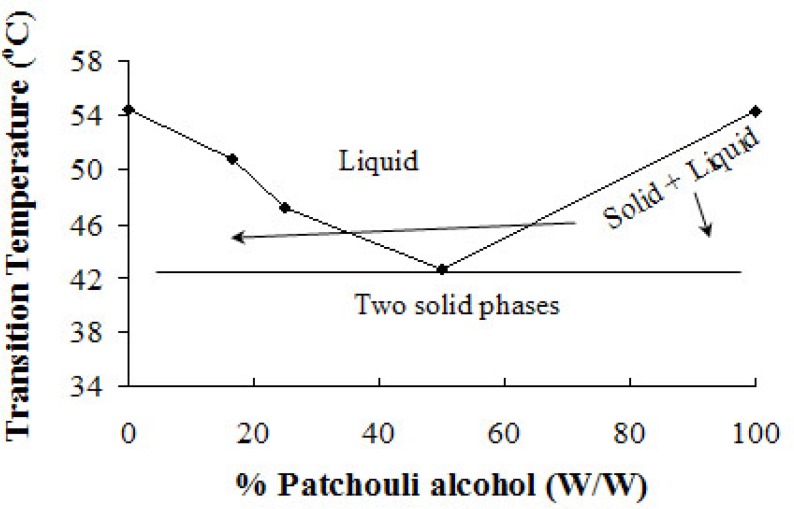
Binary phase diagram of PA/P 407 bSD pellets. The lines have no theoretical significance.

**Figure 9:**
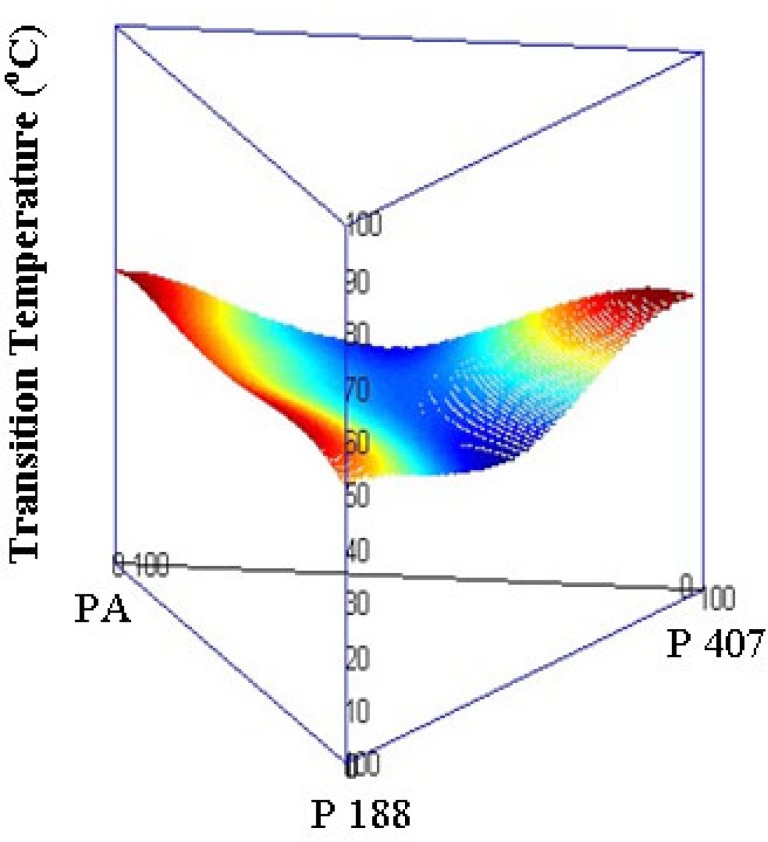
Ternary phase diagram of PA/P 407/P 188 tSD pellets.

DSC curves obtained for pure material, solid dispersions, and their corresponding physical mixtures (PM) are displayed in Figure 6. The DSC curve of PA (Figure 6Aa) exhibited a sharp endothermic peak at 54.30 °C corresponding to its melting point. In the curve of P407 (Figure 6Da), a sharp peak at 54.37 °C was observed, which was associated with the endothermic melting of P 407. The endothermic peak of P 188 appeared at 50.86 °C (Figure 6Ba). Concerning the solid dispersions, as the amount of PA increased, endothermic peaks shifted to lower temperature and then increased (Figures 7-9). While the endothermic peaks of physical mixtures of PA and poloxamers were almost unchanged irrespective of the ratios of PA and poloxamers (Figures 6B, D, F, H). The melting point of PA/P 188, PA/P 407 and PA/P 407/P 188 solid dispersions (Figures 6Ab-d, Cb-d, Ea-c, Ga-c) were lower than each pure constituent (Figures 6Aa, Ba, Da), indicating the formation of eutectic systems. When a mixture, consisting of a slightly soluble drug and an inert, highly water soluble carrier, is dissolved in an aqueous medium, the carrier will dissolve rapidly, releasing very fine crystals of the drug (18, 36). The large surface area of the resulting suspension should result in an enhanced dissolution rate and thereby improved bioavailability (37). It may be one of the mechanisms for the dissolution rate enhancement of PA-SD pellets.

*IR analysis*

**Figure 10:**
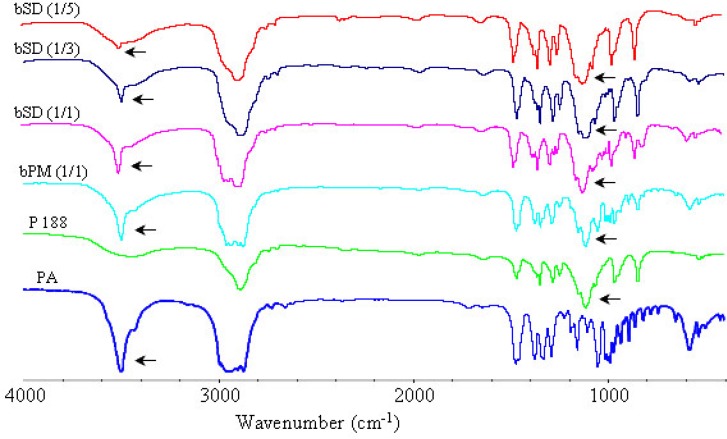
FTIR spectra of PA, P 188, physical mixture and bSD pellets

**Figure 11:**
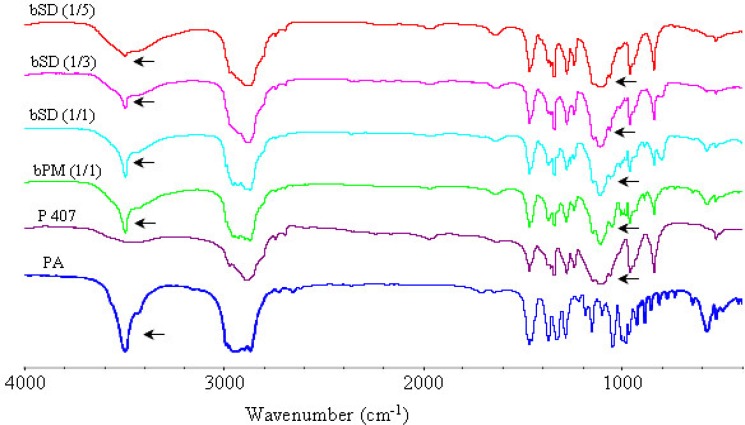
FTIR spectra of PA, P 407, physical mixture and bSD pellets.

**Figure 12:**
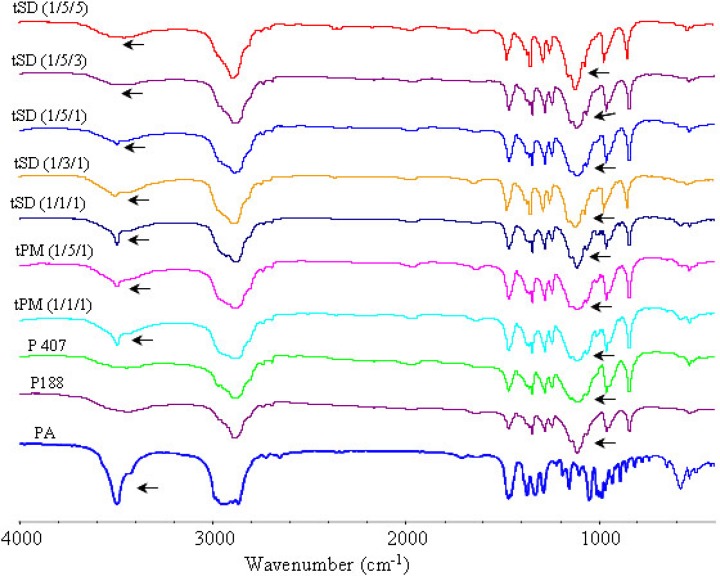
FTIR spectra of PA, poloxamers, physical mixture and tSD pellets

The FTIR spectra of pure PA, poloxamers P 188 and P 407, physical mixtures of poloxamers with PA and corresponding SDs are illustrated in Figures 10-12. The peak at 3499 cm^-1 ^was attributed to the stretching vibration of functional group of O-H of PA. The poloxamers possessed the main absorption bands at 2888 cm^−1^ and 1113 cm^−1^. The band at 2888 cm^−1^ was due to the stretching vibrations of the C-H and the band at 1113 cm^−1^ reflected the C-O group stretching.

The spectra of SDs and physical mixtures were largely similar to the addition spectra of individual components, new peak was not observed other than characteristic peaks of PA and poloxamers at FTIR spectrum of PA/poloxamers composite. This suggested that there was no chemical interaction between PA and poloxamers. But there were also very subtle differences, which could indicate the existence of intermolecular interactions between PA and poloxamers. Increasing poloxamer ratio in SDs caused weakening and shifting of the O-H stretching vibrations from 3499 cm^−1^ to 3495 cm^−1^. These subtle changes in FTIR spectra were most probably the result of formation of hydrogen bonds between PA and poloxamers. 

## Conclusion

The obtained results indicated that poloxamers P 188 and P 407 were suitable for the preparation of SD pellets with PA by the low temperature melting method. bSDs with either P 188 or P 407 had significantly increased the dissolution rate of PA, with P 188 showing a greater dissolution rate enhancement and P 407 having a better solubilization capability. As compared to the use of P 407 alone, the incorporation of P 188 in PA/P 407 bSD pellets could strongly enhance the dissolution rate of PA. The described method for pellets preparation might find application in the manufacturing and scaling-up of solid dispersion formulations in the future. Moreover, the established closed* in-vitro* dissolution test method is simple, accurate for assessing volatility drug dissolution.
